# A shorter pre-vitrification equilibration time for laser-collapsed human blastocysts is associated with a lower miscarriage rate in ART treatments

**DOI:** 10.5935/1518-0557.20250013

**Published:** 2025

**Authors:** Romualdo Sciorio, Liuguang Zhang, Yuhu Li, Ning Li

**Affiliations:** 1 Fertility Medicine and Gynecological Endocrinology Unit, Department Woman-Mother-Child, University Hospital of Vaud, CHUV, Lausanne, Suisse; 2 Center for Reproductive Medicine, Haikou Mary Hospital, Haikou 570203, China

**Keywords:** blastocyst vitrification, artificial collapse, exposure to equilibration solution, pregnancy outcomes, miscarriage rate, neonatal outcomes.

## Abstract

**Objective:**

Frozen embryo transfer in humans, especially at the blastocyst stage, provides a valid alternative to fresh embryo transfer. However, protocols for blastocyst vitrification are not yet standardized; for example, exposure to the first equilibration solution before vitrification commonly ranges from 2-3 minutes at 37°C or 2-15 minutes at room temperature. This study compared the clinical and neonatal outcomes involving vitrified-warmed blastocysts.

**Methods:**

The main aim of this prospective study was to compare the clinical and neonatal outcomes for 831 warmed blastocysts, returned in 585 frozen embryo transfers with two exposure times to the equilibration solution at room temperature: (A) 7-8 minutes and (B) 9-10 minutes.

**Results:**

The patients’ characteristics were comparable between the two groups with no significant difference in their mean age, the average number of blastocysts transferred, basal Follicle Stimulating Hormone, body mass index (BMI), infertility duration, primary infertility, or endometrial thickness. The survival and clinical pregnancy rates of the vitrified-warmed laser collapsed blastocysts were not different between the two groups. The overall miscarriage rate was significantly lower in the 7-8 minute compared to the 9-10-minute equilibration group (A: 7.6% versus B: 14.2%, p<0.05). Live birth, multiple gestation and neonatal outcomes were similar between the two groups.

**Conclusions:**

Our results indicate that the equilibration time can affect the efficiency of the cryopreservation process of human blastocysts.

## INTRODUCTION

Since the birth of Louise Joy Brown in 1978, assisted reproductive technology (ART) has been constantly increasing, and has permitted millions of infertile couples to conceive (De Geyter *et al*., 2020). Advancements in ART have been achieved thanks to several improvements, including ovarian stimulation, fertilization, embryo culture procedures and, importantly, to the progress in cryopreservation methods with the application of vitrification (Kuwayama *et al*., 2005a; 2005b). Indeed, the ability to cryopreserve human embryos has improved significantly in the last decade (Rienzi *et al*., 2017; Sciorio *et al*., 2018; Nagy *et al*., 2020). Since the introduction of minimal volume vitrification protocols, it has become increasingly evident that vitrification is very effective for human blastocysts (Ferreux *et al*., 2018; Sciorio *et al*., 2018; 2019). However, it remains important to ascertain whether the methods utilized to cryopreserve human embryos might be improved.

Cryoprotective agents (CPAs) applied during the vitrification process might negatively impact cellular metabolism and function, cell growth and apoptosis (Kollerup Madsen *et al*., 2018; Smith *et al*., 2018). Also, a high concentration of CPAs might increase the levels of reactive oxygen species (ROS) and induce epigenetic dysregulation (Zhao *et al*., 2016; Verheijen *et al*., 2019; Barberet *et al*., 2020; Lin *et al*., 2022; Mao *et al*., 2022). Several publications reported that human embryos should be kept in the vitrification solution (VS) for a maximum of 1 minute (Loutradi *et al*., 2008; AbdelHafez *et al*., 2010; Zhu *et al*., 2011), while the equilibration times normally ranged from 2 to 15 minutes (Sciorio *et al*., 2018; 2019; Verheijen *et al*., 2019; Nagy *et al*., 2009; 2020).

The exposure time of embryos to CPAs represents an important concern for successful vitrification. Longer exposure to equilibration solution (ES) may be detrimental to further embryo development, while a shorter time may be not enough for the penetration of CPAs into the cells. Contrasting results have been found in the literature, with studies reporting a fixed equilibration time of 5 minutes and others increasing the exposure time up to 15 minutes (Bagis *et al*., 2005; Hiraoka *et al*., 2009; Rezazadeh Valojerdi *et al*., 2009; Selman *et al*., 2009; Kader *et al*., 2010; Xie *et al*., 2010; Shi *et al*., 2012; Desai *et al*., 2013). Novel studies have shown that a shorter equilibration time of 8-11 minutes can be sufficient for effective vitrification of human blastocysts (Mitsuhata *et al*., 2020). Also, a study investigating blastocyst vitrification in the bovine model, by Martínez-Rodero *et al*. (2021) showed that a shorter equilibration time (3 minutes) during the vitrification procedure resulted in a significantly lower apoptosis rate when compared to a longer equilibration time (12 minutes).

These variations imply that an agreement is still missing regarding the correct equilibration time for human embryo vitrification and, therefore, to improve our vitrification protocol we decided to investigate the effects of two different equilibration times (7-8 versus 9-10 minutes) and to establish any correlation with embryo survival rate, pregnancy and clinical outcomes, and percentage of spontaneous miscarriage.

## MATERIALS AND METHODS

This was a prospective study, performed at the Centre for Reproductive Medicine, Haikou Mary Hospital, China from March 2018 to May 2022, and included 585 frozen embryo transfers (FETs). Informed consent for experimentation with human subjects was obtained before the patients started their ovarian stimulation. All participants understood what the study was and what they were consenting to and had the opportunity to opt out at any time. Female age <35 years, and causes of infertility included male factors, female infertility, and unexplained infertility. In this prospective cohort study, all patients undergoing ART treatment with embryos available for vitrification were included in the investigation, excluding only couples undergoing preimplantation genetic assessment and those azoospermic males who had to be treated with surgical sperm retrieval for oocytes injection.

Embryos were produced by either standard IVF insemination or using intracytoplasmic sperm injection (ICSI), according to the sperm assessment previously performed. Following the fresh embryo transfer, ≥2 supernumerary good quality blastocysts, according to Gardner’s score (Gardner & Schoolcraft, 1999) excluding those with a grade of CC, BC, or CB, were vitrified on day 5 after fertilization. The study included 831 expanded blastocysts, which were divided into two groups according to the equilibration time: group (A) 7-8 minutes (n=413) and group (B) 9-10 minutes (n=418). To avoid any skewing of results and to perform blastocyst allocation as equally as possible, we included in the study only those patients who had a minimum of two blastocysts available for vitrification, assigning one blastocyst to group A (7-8 minutes) and the other to group B (9-10 minutes). Exposure time in ES was meticulously controlled by an additional operator, who recorded the exact time of transfer into the vitrification solution. Also, all blastocysts with a Gardner’s expansion grade of ≥3 were artificially collapsed with a laser shot at the minimum setting. Expanding blastocysts contain a considerable amount of fluid, which may be susceptible to formation of ice crystals and increase the risk of cell death. Thus, as described by others, artificial shrinkage (AS) induces a collapse over a short time and converts the blastocyst to a morula-like stage without any fluid-filled cavity, allowing an easy passage of CPAs into the embryo (Mukaida *et al.*, 2006; Taylor *et al.*, 2010; Li *et al.*, 2014; Levi-Setti *et al.*, 2016; Sciorio *et al.*, 2018; 2019).

The primary outcomes of this study were to investigate if different equilibration times might have an impact on live birth rate (LBR) and miscarriage rate. The secondary outcomes included survival rate at warming and the clinical pregnancy rate (CPR).

### Ovarian stimulation

In all patients, controlled ovarian stimulation was achieved with either a Gonadotrophin-Releasing Hormone (GnRH) agonist (subcutaneous Buserelin 0.5 ml daily) or GnRH antagonist (subcutaneous Cetrorelix 0.5 mg daily, Merck Serono) treatment. The long protocol was used for downregulation using subcutaneous Buserelin or the GnRH antagonist; Cetrotide was administered daily on day six of the onset of menses. Ovarian stimulation was carried out using either Gonal F (Merck Serono) or Menopur (Ferring) based on individual patient characteristics. Follicular development was monitored by transvaginal ultrasound and ovulation was triggered when three follicles were 18 mm or above. Each patient received human Chorionic Gonadotrophin (hCG; Ovitrelle 0.25 mg, Merck Serono) to trigger ovulation.

### Oocyte retrieval and embryo development

Oocyte recovery was carried out under sedation using transvaginal ultrasound guidance at 36 hours following Ovitrelle injection using a 17-gauge single-lumen needle (K-OPS-7035-RWH-ET; Cook Medical Australia) (Sciorio *et al.*, 2019). Cumulus-oocyte-complexes (COC) were isolated from follicular fluid, rinsed, and transferred to 0.6 ml of Universal IVF Medium (CooperSurgical Fertility Solutions, Denmark) covered with oil for tissue culture (CooperSurgical Fertility Solutions, Denmark) in four-well dishes (Nunc™; ThermoFisher Scientific) and returned to the incubator (Astec Co., Ltd, Japan) equilibrated at 37°C, 6% CO_2_, 5%O_2_ and 89% N_2_. All media used were covered with oil and incubated overnight. Semen used for either standard IVF insemination or the ICSI procedure was collected by masturbation and processed using a standard method as reported by Bourne *et al.* (2004). All oocytes were cultured in Universal IVF Medium on the day of insemination as reported by Zhang *et al.* (2022).

The technique for insemination (standard IVF or ICSI) was decided according to the semen sample parameters and the histories of the couple. For IVF procedures, oocytes were exposed to 150,000 motile sperm/ml, while ICSI injection was performed 38-42 h post-hCG. Fertilization was identified by the presence of two pronuclei and two polar bodies approximately 16-19 hours post insemination or microinjection. At this stage, normally fertilized oocytes were cultured individually in a 25 µl pre-equilibrated droplet of Quinn’s Advantage Cleavage medium (CooperSurgical Fertility Solutions, Denmark) under oil in a standard incubator (Astec Co., Ltd, Japan) equilibrated at 37°C, 6% CO_2_, 5% O_2_ and 89% N_2_. Dishes were removed from the incubator for morphological assessment approximately 42-44 hours after insemination on day 2.

On the morning of day 3, about 66-68 hours post-insemination, embryos were moved from cleavage medium to a 25 µl droplet of Quinn’s Advantage Blastocyst medium (CooperSurgical Fertility Solutions, Denmark) and were cultured in groups of two or three embryos. On the morning of day 5, the best quality blastocyst, based on Gardner’s score (Gardner & Schoolcraft, 1999) was replaced in a fresh embryo transfer; any remaining good quality blastocysts were cryopreserved. Some patients had no fresh embryo replacement, and all the blastocysts were vitrified for future use. All blastocysts included in the study were cryopreserved on day 5.

### Artificial shrinkage of blastocysts

**Figure 1:**
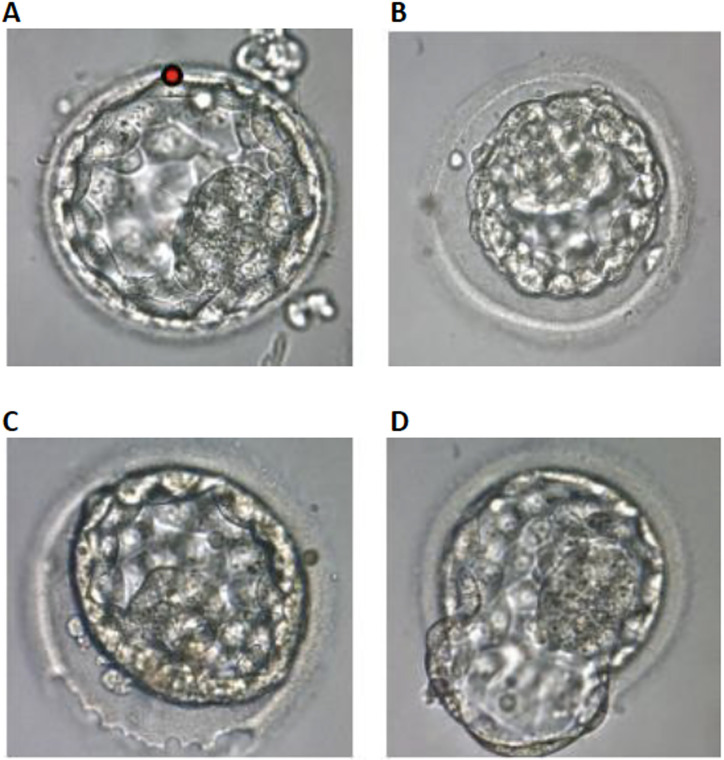
A: Laser drilling at the cellular junction of the trophectoderm before vitrification (red dot). B: Blastocyst after AS. C: A continuous laser beam was emitted tracing the ZP to drill a hole over about one quarter of the ZP surface. D: Blastocyst partly hatched from the ZP after approximately two hours culture (Magnification: x 400).

Blastocysts were classified using Gardner’s score according to blastocyst size, the morphology of the inner cell mass (ICM) and trophectoderm (TE) (Gardner & Schoolcraft, 1999). Blastocysts with a score ≥2, excluding those with a grade of CC, BC, or CB, were selected on day 5 for vitrification. Hatching and expanded blastocysts (grade ≥ 3) were artificially collapsed by applying one or two laser pulses (Hamilton Thorn Bioscience Inc, Beverly, MA, USA) before vitrification, as already reported by several authors (Vanderzwalmen *et al.*, 2002; Mukaida *et al.*, 2006; Wang *et al.*, 2007; Van Landuyt *et al.*, 2015; Levi-Setti *et al.*, 2016). The blastocyst was positioned to provide a safe distance between the ICM and the focus of the laser beam, before being exposed to a minimum setting (200 ms) laser pulse to produce a small hole at the junction of two TE cells, resulting in the discharge of fluid from the blastocoel cavity (Figure 1A). Normally, AS occurred within 1 or 2 minutes; rarely a second laser pulse was applied, and for some blastocysts responding slowly, it took up to 5-8 minutes to observe AS and disappearance of the blastocoel (Figure 1B). Subsequently, the embryo was rapidly vitrified (Sciorio *et al.*, 2019).

### Blastocyst vitrification

The Cryotop^®^ method (Kitazato Cryotop^®^, Kitazato Corporation, Shizuoka, Japan) initially described by Kuwayama and colleagues (Kuwayama *et al.*, 2005a; 2005b; Kuwayama, 2007) and utilized by several other groups (Mukaida *et al.*, 2003; Mukaida & Oka, 2012) was applied for blastocyst vitrification. The procedure comprised two different steps: equilibration and vitrification, which are both carried out at room temperature (22-25°C). The blastocyst following AS was transferred into the ES containing Trehalose functioning as an osmotic agent, which provides protection of the cellular membranes, in combination with dimethyl sulfoxide (DMSO) and ethylene glycol (EG). Given the critical point of this first step in the study, the time in ES was scrupulously measured by two operators, to establish two groups: the first in which blastocysts were kept in ES for 7-8 minutes (Group A) and the second where embryos were in ES in the range of 9-10 minutes (Group B). Those times were chosen following studies reporting that reducing the equilibration time to 8 minutes can be sufficient for successful vitrification (Mitsuhata *et al.*, 2020). Following the exposure to the ES, the embryos were exposed to the VS containing DMSO and EG, plus trehalose, gentamicin and hydroxypropyl cellulose for 45-60 seconds. Blastocysts were immediately placed on the Cryotop^®^ device, using a narrow and sterile micropipette, with the smallest possible amount of VS, and were quickly immersed into liquid nitrogen (LN_2_). A single blastocyst was always vitrified on each Cryotop^®^ device.

### Blastocyst warming and laser-assisted hatching

The Cryotop^®^ Thawing Media Kit (Kitazato Cryotop^®^) was used for warming. In a Nunc 35x10 mm culture dish, 1 ml of Thawing Solution (TS) was heated at 37°C for 30-60 minutes and then positioned on the stage warmer. The Cryotop^®^ device containing the embryo was removed from the LN_2_ and placed as quickly as possible into the dish containing the preheated TS.

The blastocyst instantly fell from the device and could be easily detected in the media under the microscope. After 1 minute, blastocysts were transferred to the Diluent Solution (DS) for 3 minutes at room temperature (22-25°C). At this stage, laser-assisted hatching (Hamilton Thorn Bioscience Inc, Beverly, MA, USA) was completed. Approximately a quarter of the zona pellucida (ZP) was thinned, using up to six laser pulses at a minimum setting (200 ms). The laser pulses were orientated on the ZP where the largest perivitelline space was seen, distant from the cells to avoid any damage (Figure 1C). However, the size of the laser hole would never exceed the thickness of the ZP, which is usually 5-10 µm in diameter. The last step was for 5 minutes, twice, in the Washing Solution (WS) and then the embryo was returned to culture in Quinn’s Advantage Blastocyst medium equilibrated at 37°C, 6% CO2, 5% O2 and 89% N2. An assessment was quickly performed on an inverted microscope to evaluate whether the embryo survived the warming, according to the morphological integrity of the ICM and TE.

After one or two hours of culture, the embryo was re-assessed again and often the re-expansion of the blastocoel was observed, which proves the embryo physiologically survived the warming procedure (Figure 1D). Embryo transfer was normally performed within 2 or 3 hours. Patients received one or a maximum of two blastocysts for transfer according to quality. In case the embryo did not survive, another embryo, if available, was warmed, otherwise the transfer was cancelled. All programmed warmed FET cycles were timed for a day-5 endometrium.

### Programmed warmed frozen embryo transfer

FETs were performed in natural and artificial cycles. Women with regular menstrual cycles (25-35 days) underwent downregulation using a GnRH agonist, Triptorelin acetate (Decapeptyl 3 mg) which was administered on day 21 of their menstrual cycle (Li *et al.*, 2022; Zhang *et al.*, 2022). Patients were instructed to take 6 mg estradiol valerate (Progynova) orally, daily from day 2 or 3 of initiation of their period after administration of Triptorelin acetate. A transvaginal scan was performed after the administration of estradiol valerate for about 14 days to measure endometrial thickness. If the endometrial thickness was more than 6 mm, embryo replacement was planned after five full days of progesterone pessary, Cyclogest 400 mg twice daily per vaginal administration. Thus, the day of embryo transfer was agreed upon after a transvaginal scan to prove that the optimal thickness was reached (preferably ≥ 8 mm). All warmed FETs were completed in a day 5 endometrium, after five full days of progesterone pessary administration. For patients who have regular menstrual cycles, as defined above, and who desired a natural cycle, baseline estradiol (E2) and Luteinizing Hormone (LH) were measured between days 2-5 of their menstrual cycle. They were then advised to have blood tests for LH/E2 from day 10 onwards either daily or every two days depending on their LH/E2 result. The blastocyst transfer was arranged four days after the detected LH peak. For luteal supplementation, progesterone vaginal gel (Crinone 8%, Merck Serono SA, Geneva, Switzerland) was utilized, which was continued daily for at least 2 weeks after embryo transfer. Serum β-hCG was measured 14 days after embryo replacement. Clinical pregnancy was determined by a fetal heartbeat on ultrasound screening after 35 days (Li *et al.*, 2022; Zhang *et al.*, 2022).

### Clinical outcome definitions

Clinical outcomes in this study were as follows: CPR, LBR, miscarriage rate, and multiple pregnancy rate (MPR). Clinical pregnancy was confirmed when a gestational sac and fetal heartbeat were visualized via an ultrasound examination at about 7 weeks of gestation; after that, any following loss of a fetus was considered a miscarriage. LBR was calculated by dividing the number of live birth deliveries by the number of transfers performed. The evaluated neonatal outcomes were as follows: sex; gestational age; birth weight; preterm birth, defined as a baby born before 37 weeks of gestation; low birth weight, defined as birth weight ≤2500 g; macrosomia, defined as birth weight ≥4000g; the delivery method; and presence of malformations.

### Statistical analysis

Data were described as numbers and percentages where appropriate. Statistical analyses were performed with either the Student’s t-test for comparison of mean values or the chi-square test to compare percentages using the Statistical Package for Social Science, version 19.0. Differences were considered statistically significant at a *p* value <0.05.

## RESULTS

A total of 831 vitrified-warmed blastocysts were analyzed in this study, of which 825 survived at the warming step (99.3%). All surviving blastocysts were transferred in 585 FETs. Overall, the CPR per transfer was 68.5%, and LBR and miscarriage rates were 61.0% and 11.0%, respectively (Table 1).

**Table 1:** Overall clinical outcomes of patients undergoing blastocyst cryopreservation.

	Overall Clinical Outcomes
Blastocyst survival	99.3% (825/831)
Clinical pregnancy	68.5% (401/585)
Live birth	61.0% (357/585)
Miscarriage	11.0% (44/401)
Multiple (twin) gestation	22.2% (89/401)

The patient’s characteristics are depicted in Table 2. There were no significant differences regarding the mean age of patients, the average number of blastocysts transferred, the basal Follicle Stimulating Hormone (FSH), body mass index, infertility duration or endometrial thickness between the two groups.

**Table 2:** Characteristics of patients undergoing blastocyst cryopreservation.

	Group A (7-8 minutes)	Group B (9-10 minutes)	*p*-value
FET cycles	285	300	
Maternal age (years)	30.17±3.48	29.50±4.09	0.767
Transferred blastocysts (n)	1.44 ±0.55	1.38±0.52	0.599
Basal FSH (mIU/mL)	7.33±1.51	7.00±1.79	0.734
Maternal BMI	22.67±2.16	21.17±2.14	0.254
Infertility duration (years)	4.17±1.47	4.67±1.75	0.604
Primary infertility	65 (26.0%)	62 (21.5%)	0.223
Endometrial thickness (mm)	8.83±1.47	9.0±1.10	0.828
Artificial cycles	244 (85.6%)	240 (80.0%)	0.073
Natural cycles	41 (14.4%)	60 (20.0%)	0.610

Results show the same survival rate after warming (group A: 99.3% and group B: 99.3%), as well as a similar CPR (A: 69.1% *versus* B: 68.0%). The LBR was slightly different between the groups (A: 63.8% *versus* B: 58.3%); this was probably due to the relatively low number of FETs performed (n=585); we are still collecting data and, presumably, when we get to more than a thousand FETs, we should reach statistical significance. Further, the multiple pregnancy rate (MPR) (A: 20.8% *versus* B: 23.5%) was comparable between the two groups. However, observing the overall miscarriage rate, data displayed a statistically significant difference (*p*<0.05) in favor of group A (7.6%) compared to group B (14.2%; Table 3). The miscarriage rate in the study period for fresh embryo replacements was 15.6%, comparable to that observed in group B.

**Table 3:** Comparison of clinical outcomes between groups A and B.

	Group A (7-8 minutes)	Group B (9-10 minutes)	p-value
Survived blastocysts (%)	99.3 (410/413)	99.3 (415/418)	1.000
Clinical pregnancy (%)	69.1 (197/285)	68.0 (204/300)	0.770
Live birth (%)	63.8 (182/285)	58.3 (175/300)	0.171
Miscarriage (%)	7.6 (15/197)	14.2 (29/204)	0.034
Multiple (twin) gestation (%)	20.8 (41/197)	23.5 (48/204)	0.513

Table 4 depicts the neonatal outcome of patients who completed the vitrified/warming program. There were no differences between the two groups concerning the parameters evaluated. The only difference observed was the percentage of low birth weight, which was higher in group B: 25.1% compared to group A: 17.0%, but this difference was not statistically significant (*p*=0.052).

**Table 4:** Comparison of neonatal outcomes between groups A and B.

	Group A	Group B	p-value
Male babies (%)	57.1 (104/182)	55.4 (97/175)	0.601
Gestational age (weeks)	38.67±1.37	38.33±1.21	0.664
Preterm birth (%)	19.2 (35/182)	20.6 (36/175)	0.733
Birth weight (Kg)	2.95±0.58	3.05±0.63	0.780
Low birth weight (%)	17.0 (31/182)	25.1 (44/175)	0.052
Macrosomia (%)	3.3 (6/182)	2.8 (5/175)	0.381
Cesarean section (%)	70.0 (128/182)	68.5 (120/175)	0.771
Congenital abnormalities (%)	0.5 (1/182)	0 (0/175)	0.913

## DISCUSSION

The results of this prospective study demonstrate that a shorter equilibration time of 7-8 minutes resulted in optimal survival, CPR, and LBR compared to exposure to ES for 9-10 minutes. Thus, this suggests that extending the duration of ES to 9-10 minutes does not bring any further benefits to the vitrification process. Above all, a longer ES time of 9-10 minutes resulted in a statistically significant higher miscarriage rate (14.2%) compared to 7-8 minutes (7.6%).

Currently, a two-step vitrification protocol is commonly adopted worldwide to cryopreserve human embryos and blastocysts (Vanderzwalmen *et al*., 2003; Roy *et al*., 2014; Sciorio *et al*., 2019; Kadour-Peero *et al*., 2022; Keshavarzi *et al*., 2022; Li *et al*., 2022; Xu *et al*., 2022). In the first solution, the ES, embryos are in contact with a lower concentration of CPAs, while in the VS they are exposed to a higher percentage of CPAs, which induces a profound volumetric change and osmotic imbalance of embryos. Limited exposure to a high concentration of CPAs is thought to be critical for the efficiency of vitrification, considering that high concentrations might induce osmotic imbalance and oxidative stress (Ginström Ernstad *et al*., 2019; Estudillo *et al*., 2021; Agarwal *et al*., 2022; Berteli *et al*., 2022; Chen *et al*., 2022; Ducreux *et al*., 2024). Therefore, a correct balance between the concentration of CPAs duration of embryo exposure is important for vitrification success (Seki & Mazur, 2012; Agarwal *et al*., 2022; Ducreux *et al*., 2024).

It is worth mentioning that temperature also plays an important role during vitrification, regulating the flow rate of CPAs into the cells (Seki & Mazur, 2012). Indeed, in this study, vitrification was performed at room temperature, using the Kitazato protocol, which suggests maintaining blastocysts in VS for 1 minute (45-60 seconds), while the time in ES generally fluctuates between 5 and 15 minutes, which agrees with several published articles (Stanger *et al*., 2012; Li *et al*., 2014; Sciorio *et al*., 2018; 2019; Endo *et al*., 2020). Animal studies have reported contrasting results on this topic. Kader *et al*. (2010) evaluated the impact of equilibration time on the DNA integrity of vitrified-warmed mouse blastocysts. They recommended an equilibration time of 8 minutes at room temperature to improve mouse blastocyst DNA integrity. Conversely, Bagis *et al*. (2005) found that vitrification with a 15 minute equilibration time resulted in a higher hatched blastocyst rate compared to that seen at 5 or 10 minutes. Recently, Berteli *et al*. (2022) analyzed about 1,000 vitrified mice oocytes and found that a longer equilibration time (10 minutes) produced lower oocyte survival and blastocyst formation rates compared to the 7-minute exposure, concluding that a longer exposure to ES might impair embryo development and cause modification in oocyte lipid composition associated with membrane integrity. Divergent results have also been found in humans, where some reports adopted a fixed equilibration time of 5 minutes (Xie *et al*., 2010; Shi *et al*., 2012), while others increase the equilibration phase pre-vitrification to 10 (Hiraoka *et al*., 2009) or up to 15 minutes (Rezazadeh Valojerdi *et al*., 2009). Xiong *et al*. (2016) analyzed this issue in 517 frozen-warmed human embryos. They split the cycles into four groups according to the equilibration time: 5-6 min, 7-8 min, 9-10 min and 11-12 min, and found no differences in terms of survival rate between the groups; but the implantation rate (IR) and LBR were lower in the 5-6 minutes exposure group compared with the three other groups. However, that study was performed on cleavage-stage embryos, while our study was performed on blastocysts, which were artificially collapsed before vitrification and therefore respond differently to the permeation of CPAs. Mitsuhata *et al*. (2020) reviewed 80 non-expanded and 112 expanded blastocysts and applied two equilibration times pre-vitrification: 8-11 and 12-15 minutes. They found no difference between the two groups in terms of survival and LBR, which agrees with our results. However, in Mitsuhata *et al*. (2020) study, the 112 expanded blastocysts were not collapsed prior to vitrification, while our study adopted a laser pulse to induce AS in all expanded vitrified embryos.

AS impacts the flow rate of CPAs into the embryo, and thus reducing the ES exposure to 7-8 minutes would be adequate to obtain an efficient vitrification process (Mukaida *et al*., 2006; Li *et al*., 2014; Levi-Setti *et al*., 2016; Sciorio *et al*., 2018; 2019). Expanding and fully expanded blastocysts enclose a considerable quantity of fluid in the blastocoel, which may increase the risk of ice crystal production during vitrification. On the other hand, when AS is applied, expanded blastocysts collapsed within a few minutes, and were rapidly converted into a morula-like stage without any fluid-filled cavity (Taylor *et al*., 2010). This is concordant with several studies applying AS prior to vitrification, reporting significant improvements in survival rate, CPR and IR (Mukaida *et al*., 2006; Taylor *et al*., 2010; Li *et al*., 2014; Levi-Setti *et al*., 2016; Sciorio *et al*., 2018; 2019).

Mukaida *et al*. (2006) showed a significant improvement in survival and pregnancy rates in 502 blastocysts using a laser pulse prior to vitrification compared to a retrospective control group. Also, Iwayama *et al*. (2011) found a significant increase in IR from 34.2% to 59.7% following AS using a laser pulse. Darwish & Magdi (2016) studied the effectiveness of AS prior to vitrification, analyzing about 500 human blastocysts. They noted that the survival rate was significantly higher in the AS group compared with the control group (97.3% *versus* 74.9%, respectively). Further, they found a significant increase in CPR and IR if AS was performed, concluding that the elimination of blastocoel fluid prior to vitrification significantly enhances clinical outcomes in vitrified-warmed blastocysts. A recent analysis correlated IR, CPR, and LBR in 1,028 consecutive warmed cycles, in which blastocysts were vitrified either with or without AS (Levi-Setti *et al*., 2016). Results found that IR, CPR, and LBR in the AS group were significantly higher (*p*<0.05) compared to the non-AS group (IR: 29.9% *versus* 23.0%; CPR: 36.3% *versus* 27.9 %; and LBR: 26.5% versus 18.1%, respectively). A study by Desai *et al*. (2008) noted that blastocysts vitrified without AS experienced more damage and degeneration compared to blastocysts with AS. Similar to our study, George *et al*. (2016) analyzed 200 FETs and found a comparable survival rate and IR between the two groups (collapsed and non-collapsed); while the CPR approached statistical significance (collapsed 50% versus non-collapsed 37%; *p*=0.06), and the abortion rate was significantly different between the two groups (collapsed 5.0% *versus* 13.0% non-collapsed; *p*<0.05). This is consistent with our study reporting that a shorter exposure to ES pre-vitrification provided a statistically significant reduction in total miscarriage rate (7-8 minutes: 7.6% *versus* 9-10 minutes: 14.2%; *p*<0.05).

A concern recently explored by several groups was whether prolonged cryo-storage after vitrification affects embryo viability, competence, and pregnancy outcomes. Several authors have investigated the duration of storage of vitrified oocytes and embryos in LN_2_ and found no change in gene expression (Huo *et al*., 2021; Canosa *et al*., 2022; Yan *et al*., 2023). No impairments were reported by Stigliani *et al*. (2015) when embryos were stored in LN_2_ for up to 6 years. Along the same lines, Yan *et al*. (2023) investigated the pregnancy outcomes following different lengths of storage (from less than 3 years up to 10 years). They found a reduced survival rate for blastocysts that were stored for longer than 6 years. Similarly, CPR and LBR were significantly decreased in blastocysts stored for more than 6 years compared with the group frozen for less than 3 years.

No difference was reported in the rates of miscarriage and ectopic pregnancy (Yan *et al*., 2023). In our study, both groups A and B had matched storage times, which were less than 16 months. The possible mechanisms by which different exposure times to ES influence the abortion rate could be explained by several reasons, such as DNA damage and fragmentation as demonstrated in an animal model study (Kader *et al*., 2010). Spindle abnormalities were observed in vitrified blastocysts compared with fresh blastocysts (Chatzimeletiou *et al*., 2012); severe changes in temperature and osmotic and oxidative stresses, as well as damage induced by exposure to a high concentration of CPAs (Agarwal *et al*., 2022; Sciorio *et al*., 2023a; 2023b; 2023c).

Additional studies, especially large-scale epidemiological reports are needed to further understand the possible implications that CPAs and cryopreservation might have on the future health of children conceived following ART. However, the current study carries the limitation that it is not a randomized controlled trial, it is a prospective observational cohort study. Also, the possibility cannot be excluded that in those couples with more than two good quality embryos, the best quality one was replaced in the fresh cycle, although attention to allocate the same quality embryo to both groups was always paid in couples with higher numbers of blastocysts.

Finally, it is worth mentioning that the manipulation skills of each embryologist may influence the overall vitrification process; however, in this study, vitrification and warming procedures were performed by only two experienced embryologists; therefore, we do believe that variations in technique between operators presumably did not influence the results.

## CONCLUSIVE REMARKS

To conclude, this prospective study shows the relationship between different ES exposure times pre-vitrification on LBR and neonatal outcomes. Our findings demonstrated that laser collapse of expanded blastocysts prior to vitrification and a shorter equilibration time of 7-8 minutes leads to a decreased miscarriage rate and a trend towards a lower percentage of low birth weight. However, there are some potential confounding factors due to the heterogeneous nature of the study sample investigated, which may impair the validity of our conclusions. Therefore, our preliminary results require further investigations with larger studies to confirm the benefit of shorter ES exposure times as a routine protocol to improve the efficacy of the vitrification process.
